# Effect of spinal-pelvic sagittal balance on the clinical outcomes after lumbar fusion surgery

**DOI:** 10.1186/s12893-023-02240-y

**Published:** 2023-11-01

**Authors:** Li-xian Tan, Xiao-kang Du, Run-min Tang, Li-min Rong, Liang-ming Zhang

**Affiliations:** 1 Department of Spine Surgery, The Third Affiliated Hospital of Sun Yat-sen University, Guangzhou, China; 2 Guangdong Provincial Center for Engineering and Technology Research of Minimally Invasive Spine Surgery, Guangzhou, China; 3 Guangdong Provincial Center for Engineering and Technology Research of Minimally Invasive Spine Surgery, Guangzhou, China; 4 Present Address: Department of Orthopedics, Dongguan Third People’s Hospital, Dongguan, China

**Keywords:** Lumbar fusion Surgery, Spinal-pelvic sagittal balance, Lumbar degenerative Diseases, Outcomes

## Abstract

**Background:**

Spinal-pelvic sagittal balance is important for maintaining energy-efficient posture in normal and diseased states.Few reports to date have evaluated the effect of spinal-pelvic sagittal balance on clinical outcomes after lumbar interbody fusion in patients with lumbar degenerative diseases (LDD).

**Methods:**

A total of 303 patients treated with posterior lumbar interbody fusion surgery for lumbar degenerative disease from January 2012 to December 2019 were enrolled in this retrospective study according to the inclusion criteria. Preoperative and postoperative spinal-pelvic sagittal parameters including pelvic incidence (PI), pelvic tilt (PT), sacral slope (SS) and lumbar lordosis (LL) of the patients were evaluated and compared. 163 patients whose postoperative PI-LL ≤ 10° were divided into the spinal-pelvic match group (Group M), while 140 patients were divided into the spinal-pelvic mismatch group (Group MM). Preoperative and postoperative Oswestry Disability Index (ODI) and Visual Analog Scale (VAS) for back pain of both groups were compared.

**Results:**

There was no significant difference between the two groups in demographic and surgical data, except for blood loss in surgery. LL, PI, PT and SS of the patients at final follow-up were all statistically different from the preoperative values in the two groups(*P* < 0.05). There was no significant difference in LL, PI, PT and SS between the two groups before surgery. At the final follow-up, LL, PI and PT differed significantly between the two groups(*P* < 0.05). Compared with the preoperative results, ODI and VAS of low back in both groups decreased significantly at the final follow-up (*P* < 0.05). Significant differences in VAS and ODI were found between the two groups at the final follow-up (*P* < 0.05). The improvement rates of VAS and ODI of Group M are both significantly higher than Group MM. Regression analysis showed that age and spinal-pelvic match had significant effects on the improvement of patients’ low back pain at the final follow-up.

**Conclusions:**

lumbar interbody fusion can significantly improve the prognosis of patients with LDD. In terms of outcomes with an average follow-up time of more than 2 years, the spinal-pelvic match has a positive effect on patients’ quality of life and the release of low back pain.

## Background

Over the past 30 years, surgeons have increasingly recognized the importance of spinal-pelvic sagittal balance in normal spinal function. Spinal-pelvic alignment balance is important for maintaining energy-saving postures in both normal and diseased states. Restoring the sagittal spinal balance is directly associated with improvement in pain and function after spinal surgery in various diseased states [1, 2]. Pelvic incidence angle (PI) is a key parameter of pelvic features.PI is relatively constant after puberty and does not vary with pelvic or spinal positioning. It directly impacts pelvic alignment, overall sagittal spinal balance and lumbar lordosis (LL). What’s more, PI is closely associated with pelvic inclination (PT) and sacral inclination (SS) parameters (PI = PT + SS), The ability of the spine and pelvis to achieve sagittal balance depends on changes in PI and other spinal-pelvic parameters [3].

Although PI is fixed, it regulates and attempts to maintain sagittal balance mainly through changes in LL. In states of greater imbalance, pelvic position may change (PT increases and SS decreases) in an attempt to maintain an upright posture. Abnormalities in spinal-pelvic parameters can lead to a variety of spinal disorders, including spondylolysis, degenerative spondylolisthesis, deformity, and compromised outcomes following spinal fusion [3, 4].

The alignment of the spine is determined by spinal-pelvic match(PI-LL ≤ 10°) [5].A retrospective analysis of systemic risk factors for adult spinal deformity showed that PI-LL mismatch and PT were related strongly to patient-reported outcomes and quality of life [6]. Pelvic incidence and other spinal-pelvic parameters are easily and reliably measured in whole-spine(lateral) radiographs [7]. Accurate assessment and measurement of these sagittal values is essential to understand their potential role in the disease process and to promote spinal-pelvic balance at the time of surgery.

In this article, we discuss surgical strategies to correct sagittal balance by exploring the correlation between spinal-pelvic sagittal parameters and clinical outcomes after lumbar fusion. This may guide spinal surgeons on whether spinal-pelvic match in the surgical plan should be assessed and restore spinal-pelvic balance in the surgery in order to obtain better clinical outcomes.

## Methods

### Selection criteria

Inclusion criteria: (1) Age and gender are not limited; (2) Chronic low back pain for more than 3 months, low back pain visual analogue scale (VAS) score ≥ 4 points, conservative treatment is ineffective, imaging data suggest lumbar degeneration, such as lumbar spondylolisthesis, lumbar spinal stenosis, severe disc herniation, etc.; (3) Receiving posterior lumbar interbody fusion surgery, no spinal cord injury, nerve injury and other complications during the treatment; (4) Complete clinical case data and follow-up data of patients; (5) Follow-up time is 2 years or more after surgery.

Exclusion criteria: (1) Patients with a history of lumbar surgery; (2) Patients with lumbar fracture, tuberculosis, tumor and other diseases; (3) Imaging data and (or) follow-up data are not complete, unable to complete the survey.

### General information

According to the inclusion and exclusion criteria, a total of 303 patients treated with posterior lumbar interbody fusion surgery for lumbar degenerative disease from January 2012 to December 2019 were enrolled in this retrospective study. The demographic data of all patients was collected, including age, gender, BMI, number of surgical segments, length of stay (LOS) and follow-up time, basic health status, the history of alcohol and smoking, etc.

### Surgical procedure

After the diagnosis of all patients was confirmed, multiple experienced spinal surgeons discussed and obtained the surgical plan. 1–2 Surgeons at the deputy director level or above performed the surgery on the patients. The surgery was performed under general anesthesia. The classic posterior lumbar fixation and fusion, pedicle screw internal fixation, laminectomy and decompression were used. During the surgery, the nerves, blood vessels, adjacent joints and joint capsules were protected, and the fusion methods were intervertebral, intertransverse, and posterolateral fusion. Postoperative antibiotics were routinely used to prevent infection, hormones were used for 3 consecutive days, patients got out of bed 3 to 5 days after surgery, and uniform waist circumference protection was performed for 3 months after surgery.

### Radiographic evaluation

**Fig. 1:**
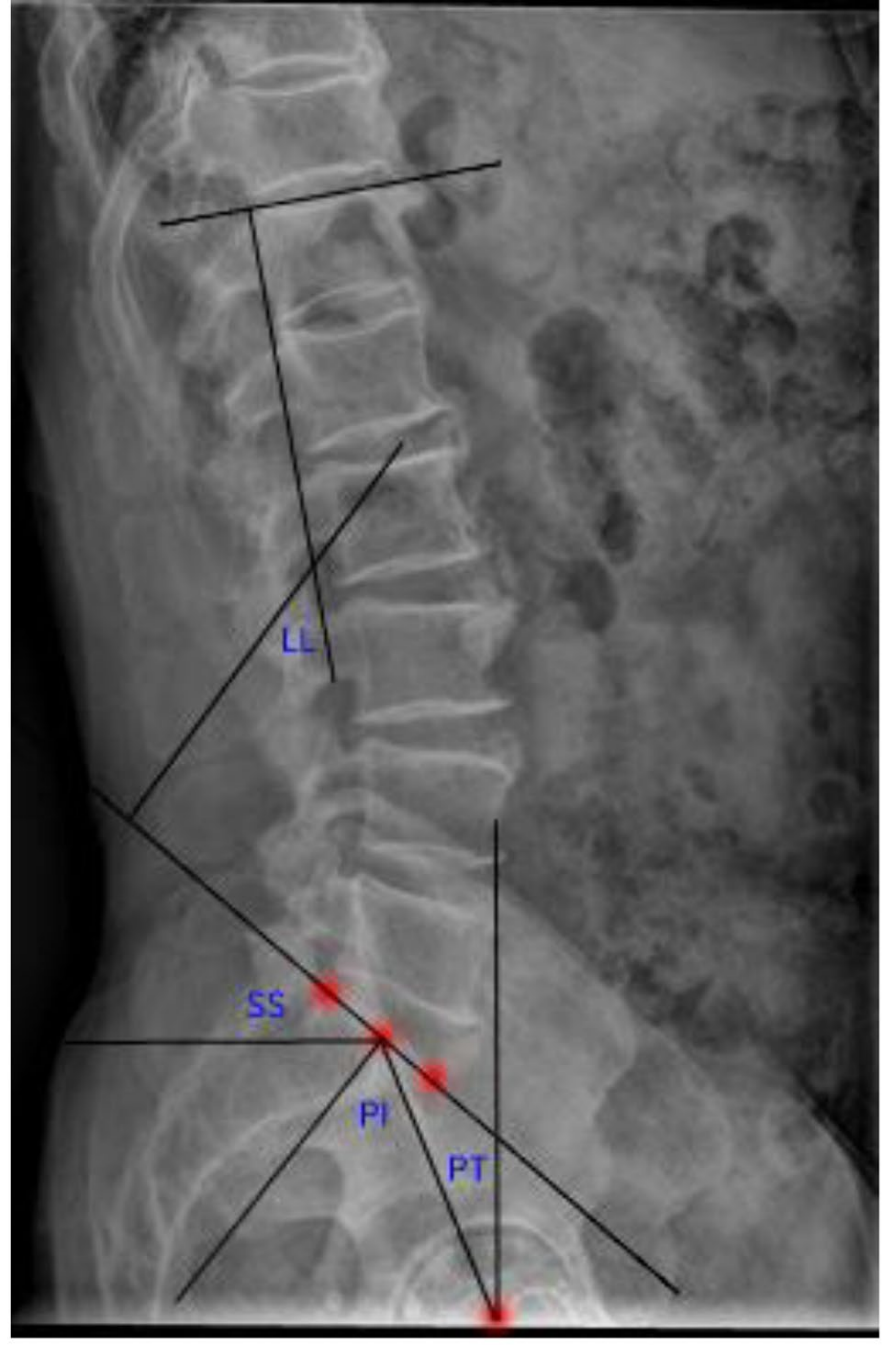
Plain lateral radiographs for measuring spinal-pelvic sagittal plane parameters. PI: Pelvic incidence; PT: Pelvic tilt; SS: Sacral slope; LL: Lumbar lordosis

**Fig. 2:**
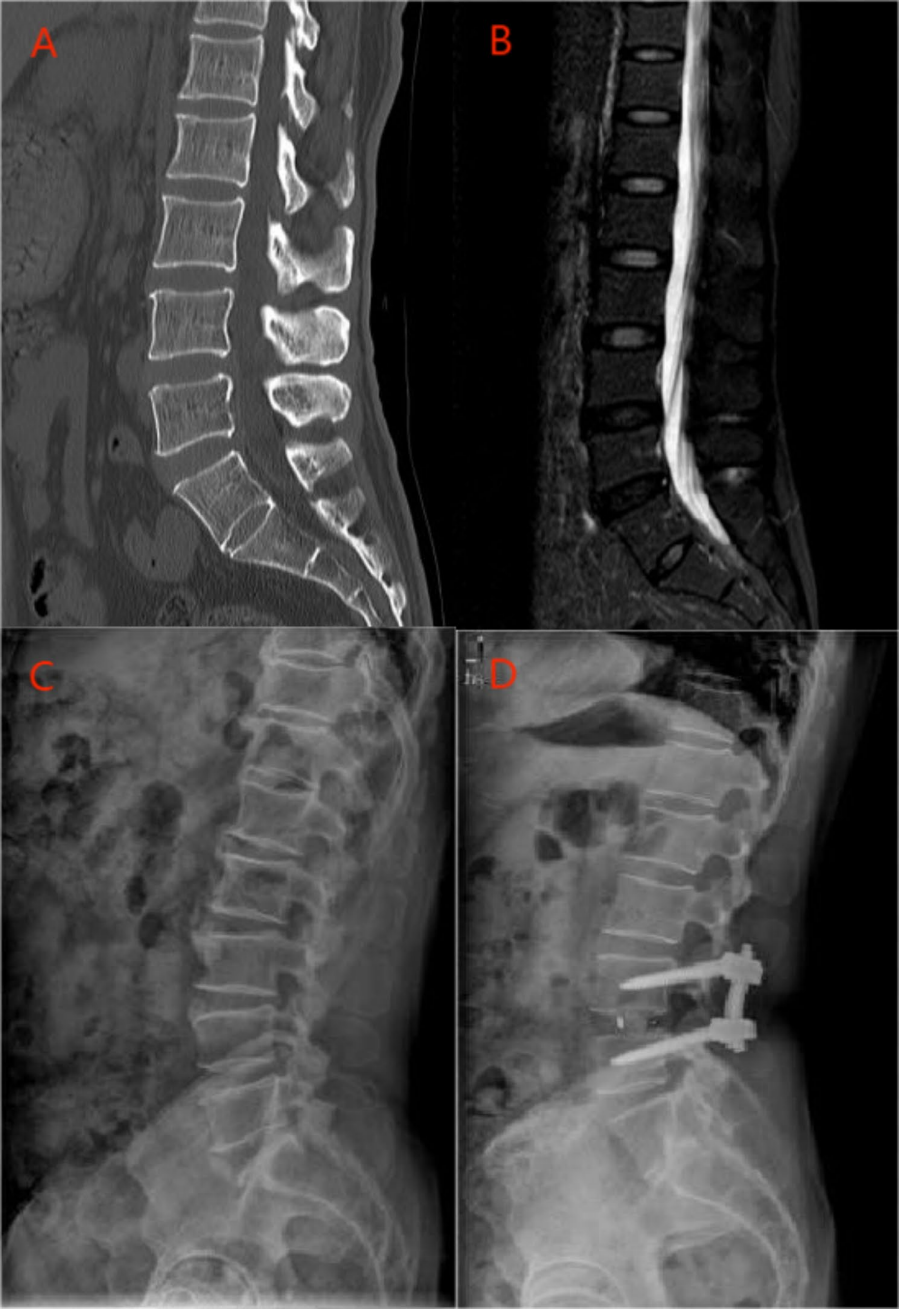
Preoperative sagittal computed tomographic scan **(A)**, sagittal T2-weighted magnetic resonance image **(B)**, sagittal lateral view **(C)**, and sagittal lateral view at the final follow-up **(D)** of a 51-year-old female patient with L4/5 degenerative lumbar spinal stenosis treated with lumbar interbody fusion

All radiological parameters were measured by three spinal surgeons (Figs. 1 and 2). In order to reduce observer bias, the assessors did not know the clinical efficacy of the patients before the assessment of imaging data. Three observers measured each radiographic parameter of the same patient twice, and the interobserver agreements were substantial, indicating that the measurement of the two observers was stable and reliable. The following radiographic parameters were measured: LL, the angle between the lines parallel to the superior endplate of S1 and the superior endplate of L1 vertebrae; SL, the angle between the upper endplate of the upper vertebrae and the lower endplate of the lower vertebrae of the responsible vertebrae; SS, the angle between the line parallel to the sacral plate and the horizontal line. PI, the angle between the line perpendicular to the midpoint of the sacral plate and the line connecting the midpoint of the femoral heads to the midpoint of the sacral plate. PT, the angle between the vertical line of the line between the midpoint of the sacral plate and the axis of the femoral heads.PI-LL ≤ 10°was defined as spinal-pelvic match and PI-LL > 10°spinal-pelvic mismatch. Spondylolisthesis is defined as the displacement of one vertebral body of the lumbar spine anteriorly or posteriorly relative to the next on a lateral lumbar spine radiograph. Lumbar instability is defined as a change in the angle between adjacent vertebral bodies of greater than 10 degrees or a relative slip of greater than 3 mm in lumbar hypertension and flexion position.

### Patients grouping

According to patients’ postoperative radiographs, 163 patients whose postoperative PI-LL ≤ 10° were divided into the spinal-pelvic match group (Group M), while 140 patients were divided into the spinal-pelvic mismatch group (Group MM).

### Functional evaluation

The patients filled out the following questionnaires before surgery and at the final follow-up, including Oswestry Disability Index (ODI) and Visual Analog Scale (VAS) for back pain. The improvement of patients’ quality of life was assessed by ODI score [8], and subjective pain perception of patients was evaluated by VAS score (0–10 score, 0 indicated no pain, 10 indicated the most severe pain) [9]. The VAS improvement rate for low back pain was defined as follows.

$$\begin{gathered}{\text{The VAS improvement rate of low back }} = \hfill \\\frac{{{\text{pre}} - {\text{op}}\,{\text{VAS}}\,{\text{of}}\,{\text{low}}\,{\text{back}} - {\text{FU VAS}}\,{\text{of}}\,{\text{low}}\,{\text{back}}}}{{{\text{pre}} - {\text{op}}\,{\text{VAS}}\,{\text{of}}\,{\text{low}}\,{\text{back}}}} \times \,100\% \hfill \\ \end{gathered}$$

### Statistical methods

SPSS 21.0 statistical software (SPSS Inc. Chicago, IL) was used for data analysis. The measurement data was expressed as mean ± standard deviation. Paired sample T test was used for comparison in the same group. Independent Sample T-Test was used for comparison in two groups. The categorical variable data was expressed as number(percentage). χ2 test was used for categorical variable data. Multiple logistic regression was used to find correlations between postoperative effects and various radiographic parameters. Correlation analysis of imaging parameters was performed using Spearman rank correlation. *P* < 0.05 was considered statistically significant.

## Results

### Demographic and surgical data

The demographic and surgical data of both groups are shown in Tables 1 and 2. Among the patients included in this study, the average age was 58.01 ± 10.96 years old and the average BMI was 24.361 ± 3.408. Female patients (178) were more than male patients (125). The single-segment fusion was more common than two-segment fusion and multiple-segment fusion in terms of the surgery. The mean age of Group M (57.99± 11.11) has no significant difference from Group MM (58.03 ± 10.82). The patients who suffered from hypertension and diabetes had no significant difference between the two groups. The average LOS was 15.29 ± 33.23 days, the average surgical time was 255.10 ± 95.25 min and the average blood loss in surgery was 248.91 ± 278.51 ml. The blood loss of Group M was significantly less than Group MM(*P* = 0.034). There were no significant differences between the two groups in terms of gender, BMI, ASA, fusion number, L4/5 fusion, L5/S1 fusion, LOS and surgical time (*P* > 0.05).

**Table 1:** Demographic data of patients in the two groups

	Full sample	Group M	Group MM	*P* value
Age	58.01 ± 10.96	57.99 ± 11.11	58.03 ± 10.82	0.978
Gender				0.599
Male	125(41.3%)	65(39.9%)	60(42.9%)	
Female	178(58.7%)	98(60.1%)	80(57.1%)	
BMI	24.36 ± 3.41	24.47 ± 3.51	24.24 ± 3.31	0.601
Alcohol	14(4.6%)	8(4.9%)	6(4.3%)	0.797
Smoking	49(16.2%)	23(14.1%)	26(18.6%)	0.293
Hypertension	95(31.4%)	52(31.9%)	43(30.7%)	0.824
diabetes	46(15.2%)	27(16.6%)	19(13.6%)	0.469

BMI: Body Mass Index

Group M: spinal-pelvic match group; Group MM: spinal-pelvic mismatch group;

**Table 2:** Surgical data of patients in the two groups

	Full sample	Group M	Group MM	*P* value
ASA				0.523
1	59 (19.5%)	28(17.2%)	31(22.1%)	
2	208 (68.6%)	116(71.2%)	92(65.7%)	
3	36 (11.9%)	19(11.7%)	17(12.1%)	
Fusion number				0.076
1	230 (75.9%)	131(80.4%)	99(70.7%)	
2	57 (18.8%)	27(16.6%)	30(21.4%)	
3	16 (5.3%)	5(3.1%)	11(7.9%)	
L4/5 fusion	237(78.2%)	122 (74.8%)	155 (82.1%)	0.125
L5/S1 fusion	78 (25.7%)	42 (25.8%)	36 (25.7%)	0.992
Surgical time/min	255.10 ± 95.25	246.78 ± 96.06	264.79 ± 93.70	0.101
Blood loss/ml	248.91 ± 278.51	215.83 ± 173.25	287.43 ± 361.62	0.034*
LOS/day	15.29 ± 33.23	14.99 ± 36.56	15.65 ± 28.98	0.863

LOS: length of stay; ASA: American society of Aneshesiologists physical status classification system; Group M: spinal-pelvic match group; Group MM: spinal-pelvic mismatch group

* Significant difference between the two groups, *P* < 0.05

### Radiographic outcomes

The radiographic parameters of patients in the full sample were shown in Table 3. LL, PI, PT and SS of the patients at final follow-up were all statistically different from the preoperative values in both groups. For the comparison between Group M and Group MM, none of the parameters differed significantly before surgery (*P* > 0.05). At the final follow-up, LL, PI and PT differed significantly between the two groups (*P* < 0.05). However the difference of SS between the two groups was not significant(*P =* 0.353).

Patients’ lumbar spine radiographs at the last follow-up showed that the rates of spondylolisthesis and lumbar instability were significantly lower than those before surgery. Either before surgery or at the final follow, there was no significant difference in the rates of spondylolisthesis and lumbar instability between the two groups. Spearman correlation analysis showed that there was a weak positive correlation between preoperative lumbar spondylolisthesis and LL (r = 0.191), PI (r = 0.149), while no significant difference was found between preoperative lumbar stability and spinal-pelvic parameters.

**Table 3:** Radiographic parameters of patients in the two groups

		Group M	Group MM	*P* value
LL (°)	PRE	41.03 ± 12.64	39.18 ± 14.08	0.229
	FINAL	47.72 ± 11.22**	41.96 ± 11.93**	< 0.001*
PI (°)	PRE	51.00 ± 11.85	53.66 ± 12.79	0.061
	FINAL	50.65 ± 11.03**	56.77 ± 11.67**	< 0.001*
PT (°)	PRE	19.94 ± 9.75	22.17 ± 10.44	0.055
	FINAL	15.42 ± 6.41**	22.52 ± 8.37**	< 0.001*
SS (°)	PRE	31.40 ± 9.53	31.40 ± 10.35	0.994
	FINAL	35.23 ± 8.78**	34.25 ± 9.32**	0.353
Lumbar spondylolisthesis	PRE	97(59.5%)	81(57.9%)	0.771
	FINAL	7(4.3%)**	5(3.6%)**	0.793
Lumbar instability	PRE	50(30.7%)	41(29.3%)	0.748
	FINAL	5(3.10%)**	3(2.1%)**	0.617

LL: lumbar lordosis; SS: sacral slope; PI: pelvic incidence; PT: pelvic tilt. PRE: preoperative, FINAL: final follow-up.Group M: spinal-pelvic match group; Group MM: spinal-pelvic mismatch group

** Significant difference compared with the preoperative, *P* < 0.05

* Significant difference between the two groups, *P* < 0.05

### Clinical outcomes

The clinical outcomes of patients in the two groups are shown in Table 4. Compared with the preoperative results, ODI and VAS at the final follow-up all decreased significantly in both groups (*P* < 0.05). In addition, significant differences in ODI and VAS were found between Group M and Group MM at the final follow-up (*P* < 0.05). The improvement rates of low back pain VAS and ODI of Group M are both significantly higher than Group MM (*P* < 0.05).

Multiple logistics regression was performed to analyze the relationship between radiological parameters and whether the low back pain was significantly improved(Improvement rate > 75%) at the final follow-up. Age, BMI, ASA, blood loss, fusion number, PI, PT, LL, SS and spinal-pelvic match at the final follow-up were included in this analysis(Table 5). The results showed that age and spinal-pelvic match had significant effects on the improvement of patients’ low back pain at the final follow-up (*P* < 0.05). Postoperative spinal-pelvic match may increase about 1.5 times likelihood(149.1%) of patients achieving significant relief of low back pain(P = 0.003).

**Table 4:** Clinical outcomes of patients in the two groups

	Group M	Group MM	*P* value
VAS of low back pain			
PRE	5.28 ± 1.10	5.29 ± 1.15	0.935
FINAL	1.09 ± 1.36**	1.61 ± 1.74**	0.004*
Improvement rate (%)	79.50 ± 24.74	69.15 ± 33.39	0.003*
ODI (%)			
PRE	18.44 ± 8.09	17.68 ± 7.06	0.386
FINAL	5.13 ± 6.64**	6.96 ± 7.79**	0.030*
Improvement rate (%)	68.93 ± 40.69	56.83 ± 55.33	0.034*

ODI: Oswestry Disability Index; VAS: Visual Analogue Scale. PRE: preoperative, FINAL: final follow-up; Group M: spinal-pelvic match group; Group MM: spinal-pelvic mismatch group

** Significant difference compared with the preoperative, *P* < 0.05

* Significant difference between the two groups, *P* < 0.05

**Table 5:** Multiple logistics regression of significant improvement of low back pain at final follow-up

	OR	95%CI	*P* value
SS	2.052	0.710, 5.930	0.184
LL	0.847	0.456, 1.575	0.600
PI	1.489	0.815, 2.719	0.195
spinal-pelvic match	2.491	1.362, 4.553	0.003*
Age	0.946	0.917, 0.976	< 0.001*
BMI	0.997	0.917, 1.084	0.945
ASA	0.689	0.389, 1.222	0.203
Blood loss	1.001	1.000, 1.0002	0.088
Fusion number	1.106	0.623, 1.961	0.731

LL: lumbar lordosis; PI: pelvic incidence; SS:sacral slope; BMI: Body Mass Index; ASA: American society of Aneshesiologists physical status classification system

* Significant difference between the two groups, *P* < 0.05

### Subgroup analysis

Subgroup analysis shows that in single-segment fusion, the improvement rate of low back pain for patients in Group M(78.95 ± 25.01) is significantly higher than that in Group MM(69.07 ± 35.04). However, in two or three-segment fusion, there is no significant difference between the two groups.

What’s more, when L4/5 or L5/S1 was performed fusion surgery, the improvement rate of low back pain for patients in Group M is significantly higher than that in Group MM.But the difference in the improvement rate for low back pain was not significant in L3/4 fusion between the two groups(Table 6).

**Table 6:** Subgroup analysis of improvement rate of low back pain between two groups in different fusion numbers and in different fusion segments

Improvement rate of low back pain(%)		Group M	Group MM	*P* value
Fusion number	1	78.95±25.01	69.07±35.04	0.018*
	2	82.09±23.56	73.59±28.65	0.229
	3	80.00±28.28	57.81±29.71	0.182
fusion segment	L3/4	80.54±21.82	69.73±30.16	0.104
	L4/5	78.96±24.33	70.87±31.38	0.028*
	L5/S1	79.69±27.56	63.13±38.14	0.034*

Group M: spinal-pelvic match group; Group MM: spinal-pelvic mismatch group

* Significant difference between the two groups, *P* < 0.05

## Discussion

Spinal degenerative diseases(LDD) are caused by gradual degeneration of the intervertebral disc with increasing age and most commonly involve the lumbar segment. [10]. Apparently, lumbar interbody fusion has been one of the routine procedures used by orthopedic surgeons to treat LDD. The biomechanical environment of lumbar interbody fusion is characterized by the presence of rigid lever arms represented by the pelvis and sacrum, adjacent to a range of lower lumbar motion segments that are more active but lordotic [11].

Spinal-pelvic-related parameters are closely related to health-related quality of life in patients with spinal deformity and degenerative disc disease [12]. At present, surgeons pay enough attention to sagittal balance, but some understanding is still not deep enough. Patients with structural spinal imbalance have predominant low back pain symptoms. Therefore, in order to evaluate the effect of spinal-pelvic sagittal balance on the postoperative outcome of patients with lumbar degenerative diseases, this study uses PI-LL and other key parameters of overall spinal sagittal balance to evaluate and analyze their correlation with postoperative low back pain.

Spinal-pelvic parameters included LL, PI, PT, and SS.PI is not affected by posture and can be used as an index to describe the shape of the pelvic and sacral orientations because the three pelvic parameters mentioned above satisfy the following formula: PI = PT + SS [3]. The spinal sagittal parameter LL can be measured by the Cobb method, which is defined as the angle between lines drawn parallel to the superior endplate of L1 and the superior endplate of S1. The criterion value for LL is approximately 46.5 ° [13].

There is a close relationship between LL and PI. Spinal-pelvic match(PI-LL) has been generated between PI and LL in recent years, which can more directly quantify the match between pelvic shape and lumbar curve, so it can be used to guide lumbar surgery planning and patients’ postoperative recovery goals [14]. One of the goals of spinal-pelvic sagittal alignment is PI-LL ≤ 10 ° threshold [15]. Recent studies have demonstrated that patients with better sagittal balance have less back pain after lumbar fusion surgery [16]. In this study, patients with spinal-pelvic match achieve higher improvement of low back pain and ODI at the final follow-up than those without. Furthermore, postoperative spinal-pelvic match may increase about 50% the likelihood(52.5%) of patients achieving significant relief of low back pain. But it’s satisfactory that all the patients who were performed with fusion surgery gained significant improvement in symptoms and quality of life. Restoration of sagittal balance after surgery has also been shown to reduce the complications of adjacent segment disease(ASD) and screw loosening, such that ASD occurs less frequently in patients with postoperative PI-LL ≤ 10 ° than in the PI-LL > 10° group [17, 18]. This may explain why patients with spinal-pelvic match have better clinical outcomes.

A study by Zhang et al. [19] showed a low ODI score and complication rate in patients with PI-LL < 10 after long-segment fusion in patients with degenerative scoliosis with a mean age of 65.1 years.Divi et al. [20] suggested that patient-reported outcomes in short-segment lumbar fusion for degenerative lumbar disease are similar in patients with and without a postoperative PI-LL mismatch. However, this study found that in single-level fusion, the improvement rate of low back pain in patients with spinal-pelvic match after surgery was significantly higher than that in those without spinal-pelvic match.On the other hand, when L4/5 or L5/S1 was performed fusion surgery, patients with PI-LL had a higher improvement rate of low back pain than those without.Because the levels of L4-5 and L5-S1 form two-thirds of LL, restoration of local and regional lordosis at these two levels is critical for preserving and improving sagittal balance [21].

PI is a relatively fixed anatomical parameter that increases progressively with age until 18 years, but essentially does not change in adulthood.PI is used as a defining pelvic position and other parameters compensate to maintain ideal sagittal alignment (PT, SS) [22, 23]. For example, LL depends on the size of the PI. When the PI value increases, both SS and LL increase compensatory and vice versa. The standard value of PI is about 53 ° ± 9 ° [24]. This study also found that the postoperative PI of Group M was closer to normal values than Group MM, which means the former has better sagittal alignment(50.63 vs. 56.77). The SS criterion is approximately 41 ° ± 8 ° [25]. In both groups of this study, the mean preoperative SS was in the normal range in both groups at the final follow-up.

PT is a characteristic of pelvic rotation and decreases as anteversion increases [22]. The standard value of PT is about 13 ° ± 6 ° [26].In both groups of this study, the mean preoperative PT was about the upper limit criterion, and PT decreased to normal values in Group M at the final follow-up, while PT did not decrease to normal values in Group MM. Some studies [27, 28] have demonstrated that improvement in PT plays an important role in sagittal reconstruction, indicating good clinical outcomes, which may explain why there was a significant recovery of postoperative PT in the significant group of patients in this study. In addition, Kim et al.(10.1186/1471-2474-12-69) [28] found that patients with PT improvement had significantly better VAS and ODI scores than patients without improvement. In our study, we found that PT in the spinal-pelvic match group is smaller than the spinal-pelvic mismatch group(15.42 vs. 22.52), which may also explain why ODI and VAS for low back pain differed between the two groups.

A previous study [29] showed that high pelvic incidence (PI) increases the risk of sagittal imbalance after spinal fusion and is a predictor of degenerative spondylolisthesis. This study also found a positive correlation between preoperative lumbar stability and LL, PI, which suggests that patients with sagittal imbalance and higher pelvic incidence have a tendency to lumbar degenerative instability.

In conclusion, lumbar fusion surgery is a clinically safe and effective treatment for LDD in lumbar degenerative diseases. With effective decompression, fixation and fusion, LDD patients are able to restore to some extent the biomechanical structures required for the spine, thereby improving their quality of life [30].In this study, we can provide strong evidence that spine surgeons should restore spinal-pelvic match as much as possible within a certain safety margin in the clinical management of patients with PLIF, which is beneficial in significantly improving patients’ postoperative low back pain symptoms. However, this is a retrospective study, which makes it difficult to avoid inaccurate patient-reported outcomes and loss of follow-up. Prospective randomized controlled studies and longer follow-up times are required to further analyze whether differences in radiographic parameters are consistent with differences in clinical outcomes.

## Conclusions

lumbar interbody fusion can significantly improve the prognosis of patients with LDD. In long-term follow-up, the spinal-pelvic match has a positive effect on patients’ quality of life and the release of low back pain. Spine surgeons should pay more attention to the sagittal balance of the patient before surgery and try to restore the sagittal balance of the patient during lumbar fusion.

## Data Availability

The underlying data supporting the results of this study could be obtained by contacting the corresponding author.

## Abbreviations

BMI: Body mass index
ASA: American Society of Anesthesiologists classification of anesthesia
LOS: Length of stay
ODI: Oswestry Disability Index
VAS: Visual analog scale
LDD: Lumbar degenerative diseases
PI: Pelvic incidence angle
PT: Pelvic tilt
SS: Sacral slope
LL: Lumbar lordosis
PRE: Preoperative
FINAL: Final follow-up
